# *Pogostemon cablin* Acts as a Key Regulator of NF-*κ*B Signaling and Has a Potent Therapeutic Effect on Intestinal Mucosal Inflammation

**DOI:** 10.1155/mi/9000672

**Published:** 2025-04-28

**Authors:** Yuqing Deng, Xin Liang, Long Zhao, Xin Zhou, Jianqin Liu, Zhi Li, Shanshan Chen, Guohui Xiao

**Affiliations:** ^1^Department of Spleen and Stomach Diseases, The Affiliated Traditional Chinese Medicine Hospital, Southwest Medical University, Lu zhou 646000, Sichuan, China; ^2^The Key Laboratory of Integrated Traditional Chinese and Western Medicine for Prevention and Treatment of Digestive System Diseases of Luzhou City, Affiliated Traditional Medicine Hospital, Southwest Medical University, Lu Zhou 646000, China; ^3^School of Integrated Traditional Chinese and Western Clinical Medicine, North Sichuan Medical College, NanChong 637100, Sichuan, China

**Keywords:** anti-inflammatory agents, chinese herbal medicine, enteritis, NF-*κ*B signaling pathway, pogostemon cablin

## Abstract

Persistent intestinal inflammation is a major contributor to various diseases, including digestive disorders, immune dysregulation, and cancer. The NF-*κ*B signaling pathway is pivotal in the inflammatory response of intestinal cells, regulating the secretion of inflammatory factors, mediating signal transduction, and activating receptors. In colitis, NF-*κ*B signaling and its effector molecules are excessively activated by various stimuli, leading to overexpression of inflammatory mediators and immune regulators. Colitis, an inflammation of the intestinal mucosa, underlies many intestinal diseases, with increasing incidence. Traditional treatments such as glucocorticoids and nonsteroidal antiinflammatory drugs have significant limitations and side effects. *Pogostemon cablin*, a traditional Chinese medicine and food, is widely used in food, spices, and pharmaceuticals. Studies have demonstrated its positive therapeutic effects on intestinal inflammation, primarily through regulation of the NF-*κ*B signaling pathway. Moreover, *P. cablin* and its active components exhibit pharmacological activities such as antiapoptotic, antioxidant, and antitumor effects. This review summarizes the original research on treating intestinal mucosal inflammation via NF-*κ*B signaling regulation using *P. cablin* and its active components, providing new insights for colitis treatment.

## 1. Introduction

Colitis is an inflammation occurring in the intestinal mucosa, caused by various factors including infections (bacterial, viral, or parasitic), autoimmune diseases (like inflammatory bowel disease, IBD), or reactions to medications, toxins, or food allergies [1]. Recent years have seen a significant increase in colitis-related diseases, particularly in China, where the prevalence of irritable bowel syndrome (IBS) and IBD is rising. Recent studies indicate that IBS prevalence in China is ~1.4% to 11.5% [2], garnering widespread research attention [3, 4]. Current effective treatments include glucocorticoids and nonsteroidal antiinflammatory drugs [5], which can alleviate symptoms and suppress inflammatory responses but have significant toxic side effects and dose dependence, making them less ideal for certain populations [6, 7]. Therefore, there is an urgent need for safer medications for colitis treatment.

*Pogostemon cablin* is a traditional Chinese medicinal herb, both edible and medicinal, indispensable in perfumes, beverages, agar, soaps, and the food industry due to its unique aroma and properties. Its diverse applications highlight its multifunctionality and vast market potential. Modern pharmacological studies show that the chemical constituents of *P. cablin* primarily include monoterpenes, triterpenes, sesquiterpenes, flavonoids, organic acids, and alcohols [8]. *P. cablin* and its active components exhibit significant antiinflammatory, antioxidant, antiapoptotic, and antitumor effects [9–11]. Studies have found that *P. cablin* and its active components significantly downregulate the protein and mRNA production of pro-inflammatory mediators such as TNF-*α*, IL-1*β*, IL-6, NO, and PGE2, inhibit the phosphorylation of IKK *β* and I *κ* B *α* and thus suppress the activation of the NF-kB pathway, demonstrating effective anti-inflammatory activity [12–19]. Additionally, the NF-*κ*B signaling pathway is one of the key mechanisms through which it exerts its antiinflammatory effects. Increasingly, studies confirm that Pogostemon cabin's inflammation control mechanisms are also applicable to intestinal inflammation regulation.

In recent years, the potential therapeutic benefits of *P. cablin* and its active components in treating colitis have gained increasing recognition. Traditional Chinese medicine (TCM) attributes the pathogenesis of colitis to damp-heat accumulation, manifesting as recurrent symptoms such as diarrhea, abdominal pain, and mucous and bloody stools. *P. cablin*, characterized by its warm nature, is linked to the spleen, stomach, and lung meridians. It functions to aromatize turbidity, harmonize the middle, and stop vomiting, primarily addressing conditions like damp turbidity obstruction, chest fullness, vomiting, abdominal pain, and diarrhea. Consequently, *P. cablin* and its active components are actively involved in reducing inflammation.

Research indicates that these natural compounds alleviate intestinal inflammation by regulating the NF-*κ*B signaling pathway. Despite this, a comprehensive review evaluating the pharmacological activities of *P. cablin*, its antiinflammatory effects on the intestines, and its regulatory mechanisms on the NF-*κ*B pathway is lacking. This study was to systematically analyze existing research on *P. cablin*'s role in treating colitis. It seeks to summarize its pharmacological material basis and mechanisms of action, particularly its effects on the direct effector molecules of the NF-*κ*B signaling pathway, thus providing new insights and directions for colitis treatment research.

## 2. Materials and Methods

### 2.1. Literature Sources

**Figure 1:**
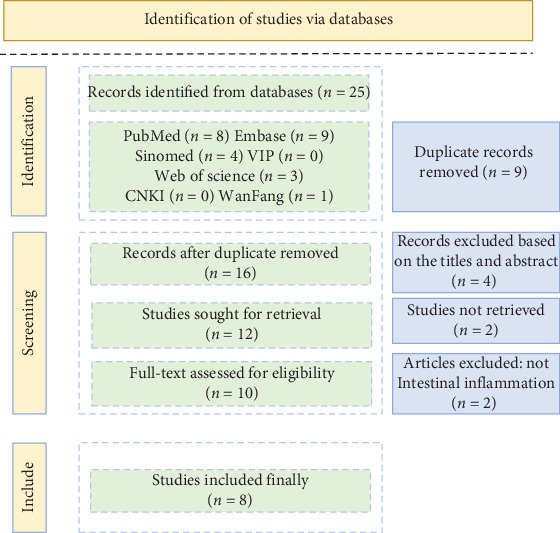
Identification of studies via databases.

This review systematically searched databases including CNKI (China National Knowledge Infrastructure), PubMed, Web of Science, Sinomed, VIP, and Embase for literature published from January 1, 2000, to April 21, 2024, evaluating research on treating intestinal mucosal inflammation via the NF-*κ*B signaling pathway using *P. cablin* (Figure 1).

### 2.2. Search Method

The search strategy included keywords and their combinations in both Chinese and English: (“GuangHuoXiang” OR “HuoXiang” OR “patchouli” OR “*P. cablin*”) AND (“YanZheng” OR “ChangDaoYanZheng” OR “intestinal inflammation” OR “inflammation”) AND (“NF-*κ*B signal pathway”). The matching was set to “fuzzy”, and manual searches were employed to avoid omissions. Literature was collected and statistics were compiled using Excel 2021.

### 2.3. Inclusion and Exclusion Criteria

#### 2.3.1. Inclusion Criteria

1. The literature must be experimental research on treating intestinal mucosal inflammation with *P. cablin* or its active components in vitro cell lines or animal models.
2. Studies must provide specific data on the pharmacological effects and actions of *P. cablin* or its active components.
3. The antiinflammatory activity of *P. cablin* and its active components must be mediated through the NF-*κ*B signaling pathway.
4. Studies must be published in peer-reviewed scientific journals.
5. Literature must be written in English or Chinese.

#### 2.3.2. Exclusion Criteria

1. Commentary articles, editorials, opinions, conference abstracts, or case reports.
2. Studies with poor methodology or incomplete reporting of results.
3. Directly related to clinical trials or case studies.

#### 2.3.3. Duplicate Publications or Secondary Literature Analysis

In the retrieved literature, we will first filter based on titles and abstracts to remove studies not meeting the inclusion criteria. Then, we will read the full texts of potentially relevant studies for further assessment.

## 3. Introduction to Patchouli

**Figure 2:**
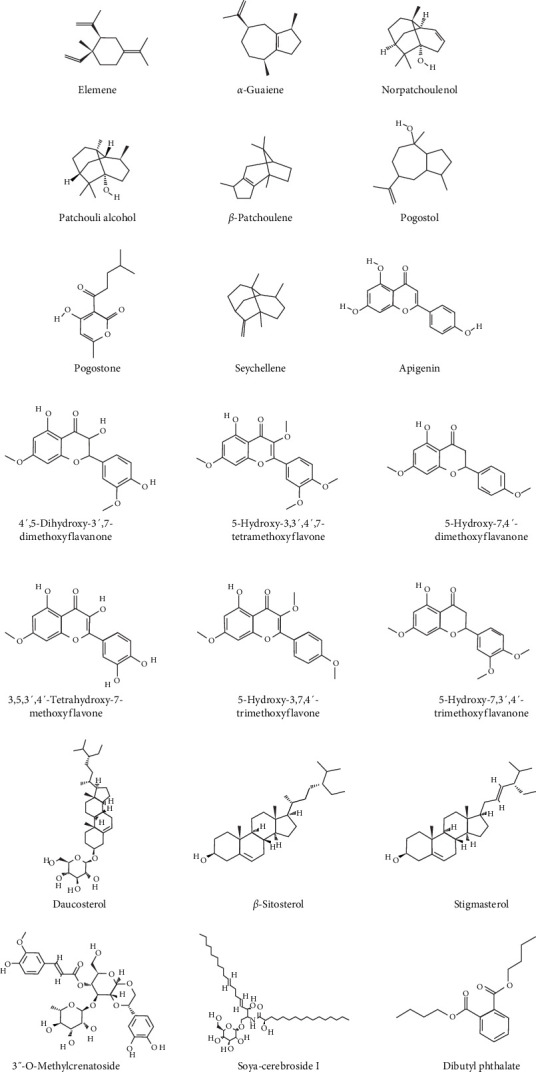
The structures of some of the *P. cablin*. chemical constituents. (All images are sourced from PubChem: https://pubchem.ncbi.nlm.nih.gov).

**Figure 3:**
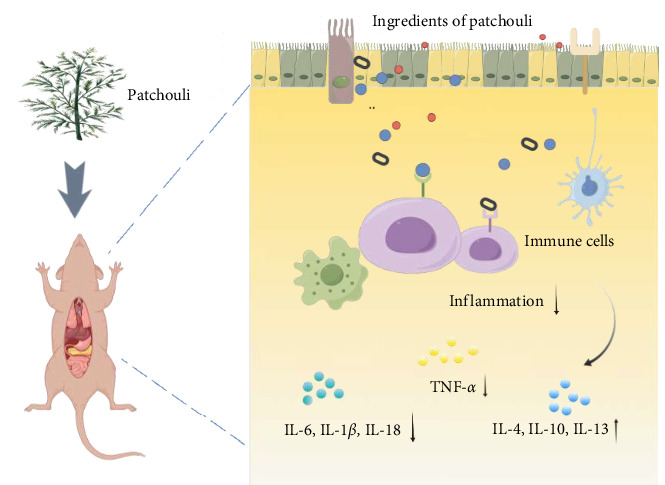
*P. cablin* can exert antimucosal inflammation effects in the intestine, and plays an important role in the treatment of intestinal inflammation(Image created by https://www.figdraw.com/#/).

Patchouli, as a medicinal name, was first recorded in the “Ming Yi Bie Lu” during the Northern and Southern Dynasties, used for treating wind-water toxin swelling, dispelling evil qi, and stopping cholera and heart pain [20]. It is primarily found in South Asian countries, including the Philippines and Indonesia. It is commercially cultivated in China, India, Indonesia, Malaysia, Singapore, West Africa, and Vietnam [21]. The 2020 edition of the Chinese Pharmacopoeia specifies that the medicinal part of patchouli is the dried above-ground parts, and the inspection items require that the leaves constitute no less than 20%, indicating that the proportion of stems and leaves significantly affects the quality of patchouli materials [21, 22]. Modern pharmacological studies have shown that the chemical constituents of patchouli mainly include terpenes, flavonoids, pyranones, steroids, and organic acids [23] (specific components as shown in Table 1, and their chemical structures are shown in Figure 2). Patchouli has been demonstrated to possess various pharmacological effects, including antiinflammatory, antiapoptotic, antioxidant, and antitumor properties [9–11]. Xu et al. [43] reported that patchouli administration resulted in a significant reduction of IL-1*β* and IL-6 levels in the colonic tissue of TgCRND8 mice. Leong et al. [44] treated C57BL/6J mice continuously for 15 days with patchouli alcohol (PA), patchoulone (PO), and *β*-patchoulene (*β*-PAE) and found that pro-inflammatory cytokines (such as IL-18, TNF-*α*) were significantly downregulated, while antiinflammatory cytokines (such as IL-4, IL-10, and IL-13) were upregulated. Furthermore, research by Li et al. [45] showed that patchouli has a protective effect on the intestinal barrier in mice with an irinotecan-induced intestinal mucositis model, able to restore mucosal damage and the expression of occludin and mucin 2 (MUC2) caused by irinotecan. In summary, patchouli has a close relationship with intestinal inflammation and plays an important role in the treatment of intestinal inflammation (Figure 3).

## 4. The Multifaceted Regulatory Mechanisms of NF-*κ*B in Intestinal Mucosal Inflammation

The nuclear factor kappa B (NF-*κ*B) system, comprising canonical and noncanonical signaling pathways, includes a family of dimeric transcription factors: p65 (RelA), RelB, c-Rel, p105/p50, and p100/p52. Typically sequestered in the cytoplasm, NF-*κ*B remains inactive due to its association with the inhibitory factor I*κ*B. External stimuli trigger the I*κ*B kinase (IKK) to phosphorylate and ubiquitinate I*κ*B, leading to its degradation and the subsequent release and nuclear translocation of NF-*κ*B. This activation enables NF-*κ*B to bind-specific DNA sequences, initiating the transcription of genes that drive the inflammatory response [46–48].

**Figure 4:**
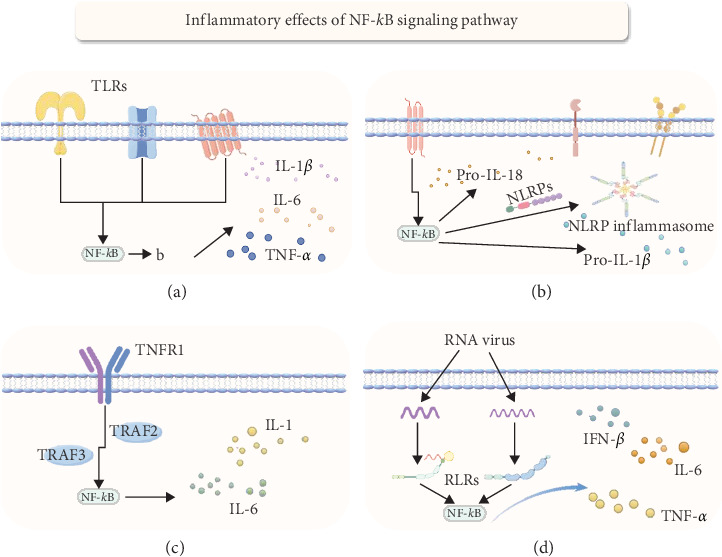
NF-*κ*B influences intestinal inflammation through different pathways. (a), When TLRs recognize pathogens or damage signals, the signaling pathways they initiate ultimately activate NF-*κ*B, activated NF-*κ*B translocates to the nucleus, promoting the expression of inflammatory cytokines such as TNF-*α*, IL-6, and IL-1*β*, thereby initiating the immune response. (b), The NLRP3 inflammasome is activated by increasing the expression of NLRP3, pro-IL-1*β*, and pro-IL-18 through the NF-*κ*B pathway. (c), TNF binds to TNFR1 and TNFR2, activating the NF-*κ*B pathway, which initiates the transcription of inflammatory cytokines, cell survival, and immune response-related genes. (d), RLRs recognize viral RNA, thereby activating NF-*κ*B to promote the expression of inflammatory cytokines (Image created by https://www.figdraw.com/#/.).

The NF-*κ*B pathway plays a crucial role in regulating several aspects of intestinal inflammation, including maintaining intestinal epithelial integrity, mediating inflammatory responses, and activating immune cells such as antigen-presenting cells and effector leukocytes [49–52]. Over-activation of NF-*κ*B during intestinal inflammation results in heightened expression of pro-inflammatory cytokines like TNF-*α*, IL-1*β*, and IL-6, perpetuating inflammation [53, 54]. This excessive activation also promotes apoptosis and damages intestinal epithelial cells, intensifying inflammation severity and fostering leukocyte migration and adhesion, which accumulates inflammatory cells in the mucosa (Figure 4).

### 4.1. NLRP

The NOD-like receptor family pyrin domain containing (NLRP) is involved in intracellular inflammation and immune responses by forming protein complexes known as inflammasomes [55, 56]. Inflammasomes are cytoplasmic multiprotein complexes, including members like NLRP1, NLRP3, NLRP6, NLRP7, NLRP12, NLRC4, and NAIP. These complexes activate caspase-1, promoting the maturation and secretion of inflammatory cytokines [57, 58].

The relationship between inflammasomes and NF-*κ*B activation is extremely complicated. The NLRP3 inflammasome, composed of NLRP3 protein, ASC, caspase-1, and NEK7, is promoted by various stimuli that activate NF-*κ*B to facilitate the transcription and expression of NLRP3, pro-IL-1*β*, and pro-IL-18[59, 60]. Existing research has shown that the expression of the NLRP3 gene is closely linked to susceptibility to inflammatory bowel diseases like Crohn's disease [61]. Furthermore, NF-*κ*B p65 and SETDB1 can activate IRF7, stimulating M1 polarization of macrophages and enhancing LPS-induced inflammation in mice through the NLRP pathway [62]. Notably, studies by Eran Elinav and others have found that mice lacking NLRP6 show increased susceptibility to spontaneous intestinal proliferation and inflammatory cell recruitment, with significantly worsened colitis symptoms [63]. Although evidence suggests that NLRs may exacerbate intestinal inflammation via NF-*κ*B signaling, some studies indicate the opposite, thus, the specific impact mechanisms in this area necessitate further exploration.

### 4.2. Toll Receptor

Toll-like receptors (TLRs) are a class of transmembrane proteins that regulate innate and adaptive immune responses through pathogen-associated molecular patterns (PAMPs), damage-associated molecular patterns (DAMPs), and other exogenous molecular patterns (xAMPs) [64]. Research has found that dysregulation of TLR signaling increases susceptibility to colitis and tumors [65]. Although activation pathways differ among TLRs, most can activate the canonical NF-*κ*B signaling pathway, promoting inflammatory responses and immune participation [66]. In patients with active ulcerative colitis (UC), the gene expression of TLR5, TLR8, and TLR9 is upregulated, with their mRNA levels positively correlated with the severity of intestinal inflammation and the expression of NF-*κ*B-induced inflammatory cytokines [50]. Additionally, a meta-analysis shows significant associations between polymorphisms in TLR1 rs5743611, TLR4 rs4986790, TLR4 rs4986791, and TLR6 rs5743810 and the risk of inflammatory bowel disease (IBD) [67]. Research also finds that the heat shock protein HSP-W reduces intestinal barrier permeability and alleviate intestinal barrier damage by upregulating the expression of tight junction (TJ) proteins through the TLR4/MyD88-mediated NF-*κ*B and p38 MAPK signaling pathways [68]. Overall, TLRs play a complex and dual role in the intestines. TLRs, therefore, play a complex and dual role in intestinal health. While their specific molecular mechanisms in colonic pathology remain incompletely understood, their key role in intestinal inflammation via the NF-*κ*B signaling pathway is well recognized.

### 4.3. RLRs

RIG-I-like receptors (RLRs) are essential sensors of microbial nucleic acids within the cytoplasm, including the main members: retinoic acid-inducible gene I (RIG-I), melanoma differentiation-associated gene 5 (MDA5), and laboratory of genetics and physiology 2 (LGP2) [69, 70]. Upon recognizing microbial RNA, these receptors activate the mitochondrial antiviral signaling protein (MAVS), triggering signaling that recruits Ser/Thr kinases, TANK-binding kinase 1 (TBK-1), I*κ*B kinase-*ɛ* (IKK*ɛ*), and IKK*α*/*β*/*γ*. These kinases activate interferon regulatory factor 3 (IRF3), interferon regulatory factor 7 (IRF7), and nuclear factor *κ*B (NF-*κ*B), inducing cytokine gene expression [71, 72]. Studies have found that mice lacking the Rig-I gene (Rig-I-/-) are more susceptible to dextran sulfate sodium (DSS)-induced colitis [73]. Additionally, commensal viruses maintain intestinal homeostasis via the Rig-I signaling pathway [74]. Mice lacking the MAVS gene (MAVS-/-) exhibit significantly increased expression of interleukin 6 (IL-6), interleukin 1*β* (IL-1*β*), tumor necrosis factor *α* (TNF-*α*), and interferon *β* (IFN-*β*) in colonic tissue, showing high sensitivity to colitis [75]. These findings indicate a significant role of RLR dysfunction in inflammatory bowel disease pathogenesis. However, further study is required to understand the significance of NF-*κ*B factors triggered by these immune sensors in intestinal inflammation.

### 4.4. TNF

The TNF superfamily (TNFSF), comprising 19 ligands and 29 receptors, plays a key role in inflammation, immune response, and cell apoptosis [76]. TNFSF members primarily signal through the NF-*κ*B signaling pathway, with most members activating NF-*κ*B via I*κ*B*α* degradation. Among the known TNF receptor-associated factors (TRAF), TRAF2, TRAF5, and TRAF6 have been proven to mediate the activation of NF-*κ*B[77]. An in vitro study showed that TNF stimulation of colonic epithelial Caco-2 cells downregulated the expression of the intestinal barrier-associated protein zonula occludens-1 and increased the permeability of Caco-2 TJs in a concentration and time-dependent manner. Specifically, researchers suppressed TNF-*α*-induced NF-*κ*B activation using the NF-*κ*B inhibitor curcumin and a triterpenoid compound from *Tripterygium wilfordii*, preventing the increase in Caco-2 TJ permeability, confirming the necessity of NF-*κ*B activation for TNF-induced permeability [78]. However, studies have noted that the TNFSF molecule TNFSF14 (LIGHT) plays an essential role in preventing severe disease in a mouse model of colitis, and the absence of the lymphotoxin *β* receptor (LT*β*R) exacerbate the onset of colitis [79]. These studies reveal the complex and bidirectional role of TNFSF in regulating intestinal inflammation. Despite primarily signaling through different pathways to activate the NF-*κ*B system, the specific immune activation mechanisms of TNFSF in intestinal inflammation require further clarification.

## 5. Mechanism of Action of Patchouli on the NF-*κ*B Signaling Pathway

### 5.1. Upstream Regulatory Mechanisms

Patchouli extracts exert systemic modulation of the inflammatory pathway by targeting multiple upstream signaling molecules within the NF-*κ*B pathway. Studies have demonstrated that one of the key mechanisms of patchouli alcohol (PA) is its ability to directly bind to and stabilize ubiquitin-specific protease 18 (USP18), leading to a significant upregulation of its expression. USP18 serves as a critical negative regulator of both the NF-*κ*B pathway and the NLRP3 inflammasome by inhibiting the recruitment efficiency of tumor necrosis factor receptor-associated factor 6 (TRAF6) within the signaling complex. This results in the attenuation of IKK complex activation, particularly through the suppression of IKK*β* phosphorylation, and the inhibition of NLRP3 inflammasome assembly [80]. Furthermore, patchoulene epoxide (PAO) inhibits the phosphorylation of IKK*β* and I*κ*B*α*, thereby reducing the ubiquitin-mediated degradation of I*κ*B*α* and sustaining the cytoplasmic retention of the NF-*κ*B complex [17]. Regarding MAPK pathway regulation, pogostone (PO) selectively suppresses the phosphorylation of p38 MAPK and JNK [18], while PA blocks ERK1/2 phosphorylation, thereby mitigating ERK-mediated NF-*κ*B activation [81]. Notably, *β*-patchoulene epoxide (*β*-PAE) and PO also activate the Nrf2 antioxidant pathway, promoting nuclear translocation of Nrf2 and upregulating the expression of antioxidant genes such as NQO-1, HO-1, and GCLC, thereby establishing a dual regulatory mechanism for oxidative stress and inflammation [14, 82]. The synergistic interactions of these upstream molecular targets provide a mechanistic foundation for the multidimensional antiinflammatory effects of patchouli extracts through the NF-*κ*B signaling pathway.

### 5.2. Targeted Intervention in the Core Pathway

Patchouli extracts exert core regulatory effects on the NF-*κ*B signaling pathway by modulating IKK/I*κ*B*α* degradation and p65 nuclear translocation. Patchoulene epoxide (PAO) directly inhibits IKK*β* phosphorylation, reducing I*κ*B*α* phosphorylation and subsequent ubiquitination-mediated degradation, thereby stabilizing I*κ*B*α* protein levels and preventing the release of the NF-*κ*B (p50/p65) complex [17]. In lipopolysaccharide (LPS)-stimulated RAW264.7 macrophages and TNF-*α*-induced HT-29 colorectal cancer cells, patchouli alcohol (PA) further inhibits NF-*κ*B activation through a dual mechanism: on the one hand, it stabilizes I*κ*B*α* protein levels by preventing its ubiquitination and degradation, and on the other hand, it blocks the nuclear translocation of the p65 subunit, effectively suppressing the downstream amplification of the NF-*κ*B signaling cascade [81]. Furthermore, patchouli ketone (PO) suppresses LPS-induced I*κ*B*α* phosphorylation and p65 nuclear translocation, further confirming the broad regulatory potential of patchouli-derived compounds in targeting the NF-*κ*B core pathway [18].

### 5.3. Transcriptional Inhibition of Downstream Inflammatory Effector Factors

Patchouli extracts significantly reduce the expression and release of pro-inflammatory mediators by inhibiting the activation of the NF-*κ*B and MAPK pathways. In an H1N1 virus infection model, patchouli alcohol (PA) suppresses NF-*κ*B signaling, thereby reducing the production of cytokines such as IL-1*β*, IL-6, and TNF-*α*, while also blocking NLRP3-GSDMD-mediated pyroptosis, alleviating virus-induced excessive inflammatory responses [80]. Notably, patchouli ketone (PO) exhibits specificity in inflammasome regulation. In a nonalcoholic fatty liver disease (NAFLD) model, PO demonstrates a strong binding affinity to NLRP3, suggesting that it may exert its effects through direct inhibition of NLRP3, thereby suppressing the production of IL-1*β*, IL-6, and TNF-*α*. This effect is significantly attenuated in NLRP3-knockout mice, confirming its target specificity. Collectively, these downstream effects mitigate the amplification of inflammatory cascades and reduce tissue pathological damage [83].

### 5.4. Dynamic Equilibrium of a Multipathway Synergistic Network

The antiinflammatory advantage of patchouli extracts is also reflected in their interactive regulation of the NF-*κ*B, MAPK, and Nrf2 pathways. First, patchouli alcohol (PA) inhibits ERK1/2 phosphorylation, thereby blocking the ERK-NF-*κ*B cascade [81], while patchouli ketone (PO) suppresses the phosphorylation of p38 MAPK and JNK, exerting dual suppression on both the NF-*κ*B and MAPK signaling pathways [18]. Second, *β*-patchoulene epoxide (*β*-PAE) and PO activate the Nrf2 pathway, upregulating HO-1 and NQO-1, thereby exerting antioxidative effects that counterbalance NF-*κ*B inhibition, forming an “oxidative stress-inflammation” regulatory axis, which has demonstrated significant protective effects in LPS-induced acute lung injury [14, 82]. Furthermore, PA inhibits both NF-*κ*B and the NLRP3 inflammasome through a USP18-dependent mechanism [80], highlighting the multitarget synergy characteristic of natural products. This network-based regulation not only enhances antiinflammatory efficacy but also reduces the risk of compensatory pathway activation associated with single-target inhibition, offering new therapeutic insights for the treatment of inflammatory diseases.

Patchouli extracts establish a multitiered antiinflammatory network through upstream regulation, core pathway blockade, and downstream suppression of inflammatory mediators. This mechanism is markedly distinct from the single-target mode of action observed in traditional nonsteroidal antiinflammatory drugs (NSAIDs), demonstrating a high-efficiency, low-toxicity, and multitarget synergistic advantage.

## 6. Pogostemon Cablin Treatment of Intestinal Inflammation via the NF-*κ*B Signaling Pathway

### 6.1. Patchouli Alcohol

Patchouli alcohol (PA) demonstrates significant antiinflammatory activity in models of intestinal inflammation. In a TNBS-induced colitis mouse model, PA modulates the serum levels of pro-inflammatory cytokines (TNF-*α*, IFN-*γ*, IL-1*β*, IL-6, and IL-17) and decreases the mRNA expression of iNOS, TNF-*α*, COX-2, IL-1*β*, and IL-6. PA's mechanism involves the inhibition of I*κ*B*α* and p65 phosphorylation, thereby blocking the NF-*κ*B signaling pathway and reducing intestinal inflammation [84]. Furthermore, PA acts as an agonist of the Pregnane X Receptor (PXR) in vitro. In a DSS-induced colitis mouse model, PA attenuates NF-*κ*B activity and nuclear translocation by activating PXR, highlighting the critical interplay between PXR and NF-*κ*B in colitis treatment. PA effectively inhibits NF-*κ*B promoter activity and transcription of its downstream targets [85]. Additionally, in a 5-fluorouracil-induced colitis mouse model, PA decreases levels of TNF-*α*, IL-1*β*, IL-6, and myeloperoxidase (MPO), while enhancing antiinflammatory IL-10 expression. This action correlates with the suppression of TLR2 and MyD88 protein expression, suggesting PA's role in inhibiting mucosal inflammation via the TLR2/MyD88/NF-*κ*B pathway [86]. In a rat model of intestinal inflammation induced by enterogenic lipopolysaccharide (LPS), patchouli alcohol (PA) demonstrated a dose-dependent antiinflammatory effect. This was evidenced by the reduction in the expression levels of CD14, MD2, MyD88, TLR4, and p- NF-*κ*B p65. Additionally, PA mitigated the increase in inflammatory markers such as tumor necrosis factor-alpha (TNF-*α*), interleukin-1 beta (IL-1*β*), interleukin-6 (IL-6), and myeloperoxidase (MPO), indicating its potential therapeutic value in modulating inflammatory responses [87]. Furthermore, PA inhibits the overexpression of iNOS and IL-6 in human colonic epithelial cells stimulated by TNF-*α*, preventing I*κ*B-*α* degradation and p65 translocation, thereby suppressing NF-*κ*B transcriptional activity [81].

### 6.2. Patchouli Oil

In a rat model of 5-fluorouracil (5-FU)-induced colitis, Patchouli Oil (P.oil) significantly mitigates the levels of TNF-*α*, IFN-*γ*, IL-13, MyD88, IRAK-4, FADD, TAK-1, phosphorylated p38, phosphorylated p65, and MPO, indicating an inhibitory effect on the NF-*κ*B and MAPK signaling pathways. P.oil achieves this by reducing p65 and p38 phosphorylation, thus alleviating inflammation [88].

### 6.3. Patchoulene

Studies indicate that the levels of TLR4 and MyD88 are significantly increased in UC mouse models. However, *β*-PAE markedly reduces MPO, TNF-*α*, ICAM-1, and P-selectin levels, alongside TLR4 and MyD88 expression. Further investigation reveals that *β*-PAE inhibits I*κ*B*α* phosphorylation and p65 nuclear translocation, processes that are notably enhanced in colitis models [89].

### 6.4. Water Extract

Research demonstrates that the water extract of Pogostemon Cablin (PCW) alleviates TNBS-induced colitis by inhibiting NF-*κ*B-dependent pro-inflammatory cytokines. Specifically, PCW decreases the binding of monocytes to HT-29 human colonic epithelial cells stimulated by TNF-*α* and shows dose-dependent inhibition of colitis markers such as weight loss, MPO activity in colon tissue, and COX-2 expression. PCW also downregulates MCP-1, IL-8, and IL-6 mRNA levels in rat colons induced by TNBS. In vitro, PCW significantly inhibits NF-*κ*B luciferase reporter gene activity in HT-29 cells treated with TNF-*α*, underscoring its role in colitis management through the suppression of NF-*κ*B-dependent pro-inflammatory cytokine expression [90] (The summarized information pertaining to each individual component has been comprehensively compiled in Table 2).

## 7. Conclusion and Discussion

*P. cablin*, renowned for its distinctive aroma and medicinal properties, has been extensively utilized in traditional medicine for millennia. Despite its long history, contemporary research into its active components—such as patchouli alcohol, patchoulene, patchoulone, and patchouli epoxide—and their effects on the NF-*κ*B signaling pathway is relatively recent. Advances in scientific research have intensified interest in these compounds for their potential to mitigate intestinal inflammation.

This review elucidates the mechanisms by which *P. cablin* and its active constituents modulate the NF-*κ*B signaling pathway to combat intestinal mucosal inflammation. Studies indicate that *P. cablin* functions as an antiinflammatory agent by downregulating pro-inflammatory cytokines and inhibiting the phosphorylation of p65 and I*κ*B*α*. For example, patchouli alcohol (PA) has demonstrated significant antiinflammatory effects in animal models, where it reduces inflammatory cytokine levels, ameliorates histological disturbances in colitis models, and decreases neutrophil infiltration [84]. Additionally, *β*-patchoulene ester (*β*-PAE) has been shown to protect tight junctions in the colons of ulcerative colitis (UC) mouse models, reduce neutrophil aggregation, and inhibit colonic inflammation through the TLR4/MyD88/NF-*κ*B signaling pathway [89]. Patchouli oil similarly downregulates the expression of TLR2, TLR4, TNF-*α*, IFN-*γ*, IL-13, and MPO, and significantly inhibits proteins associated with the TLR-MyD88 pathway [88].

*P. cablin* exhibits potent antiinflammatory effects even at low doses, making it a promising candidate for treating intestinal inflammation. Animal model studies reveal that various components of *P. cablin* effectively alleviate disease symptoms, improve intestinal pathology, and suppress inflammatory responses. Notably, the efficacy of *P. cablin* compares favorably with standard antiinflammatory drugs. For instance, Wu et al. [84] reported that the efficacy of patchouli alcohol is comparable to sulfasalazine (SASP). Furthermore, *β*-patchoulene ester (*β*-PAE), particularly at lower doses (5, 10, and 20 mg/kg), outperforms SASP and offers a better safety profile due to its lower toxicity (LD50 > 10 g/kg) [89]. This makes *β*-PAE a preferable treatment option, given the known liver and kidney toxicity of SASP. Additionally, *β*-PAE not only alleviates symptoms of UC but also provides protection against liver injury, thereby reducing potential side effects.

The safety and low toxicity of *P. cablin*'s components highlight their therapeutic potential. Current research predominantly focuses on patchouli alcohol, a major active component of patchouli oil and a sesquiterpene compound with known antiinflammatory, antibacterial, and antioxidant properties. The antiinflammatory effects of patchouli alcohol may be attributed to its lipophilic nature and specific molecular structure, which enable it to bind to biomolecules such as cyclooxygenase (COX), influencing multitarget disease outcomes. While these mechanisms are promising, further research is necessary to fully elucidate the therapeutic potential and mechanisms of action of patchouli alcohol.

*P. cablin* is rich in bioactive compounds such as patchouli alcohol (PA) and patchoulene, the contents of which are influenced by multiple factors, including geographical origin and extraction methods. In recent years, the extraction techniques for patchouli's active components have diversified, encompassing traditional methods such as hydrodistillation and steam distillation, as well as emerging technologies like supercritical CO_2_ fluid extraction, microwave-assisted extraction, and membrane separation. These techniques have been systematically applied to enrich its essential oil and functional constituents [91–93]. Studies have demonstrated significant compositional differences in patchouli extracts obtained via different extraction methods, particularly in the content of phenolic and terpenoid compounds [94]. Beyond extraction methods, geographic location plays a crucial role in determining the chemical composition of patchouli. Research reports indicate variations in the volatile oil content of *P. cablin* cultivated in different regions of China, such as Guangzhou, Zhaoqing, Zhanjiang, and Hainan. Certain cultivars, such as *P. cablin* cv. Shippai (Paixiang), are considered to be of superior quality. Chemical analyses have identified 31, 33, and 42 volatile compounds in the Paixiang, Zhaoxiang, and Zhanxiang cultivars, respectively [23]. The physicochemical differences among extraction techniques can alter the bioactive composition of patchouli, thereby influencing its pharmacological activity. Studies have shown that the essential oils obtained via different extraction methods exhibit significant variations in nitric oxide (NO) inhibition activity. Among these, patchouli oil extracted using supercritical CO_2_ extraction demonstrated the highest NO inhibitory capacity, with an average inhibition rate exceeding 75%. In contrast, patchouli oil obtained via petroleum ether cold maceration exhibited a moderate inhibition rate of ~60%, while that extracted through steam distillation had relatively weaker activity, with an average inhibition rate below 50% [95]. These findings suggest that the antiinflammatory potential of patchouli may vary depending on the extraction process. However, direct evidence linking these compositional differences to overall antiinflammatory efficacy or potential adverse effects remains limited. Future studies should integrate multidimensional pharmacodynamic evaluations and mechanistic investigations to comprehensively assess the impact of geographical origin and extraction methods on the bioactivity of patchouli.

## Figures and Tables

**Table 1:** The constituents of *P. cablin*.

The volatile constituents of *P. cablin*				
Compound name	Formula	Plant part	Analytical method	References
*α*- and *β*-Bulnesene	C_15_H_24_	Leaves (hydro-distillation extracts)	GCMS;NMR	[24–26]
*α*-Caryophyllene	C_15_H_24_	Leaves (hydro-distillation extracts)	GCMS;NMR	[26–31]
*α*-, *β*- and *δ*-Elemene	C_15_H_24_	Leaves (hydro-distillation extracts)	GC;GCMS;NMR	[25, 26, 28]
*α*-, *β*- and *δ*-Guaiene	C_15_H_24_	Stem and leaves (methanol extracts)	GC;GCMS;NMR	[27–32]
Norpatchoulenol	C_14_H_22_O	Leaves(steam distillation extracts)	GC	[28, 30]
Patchouli alcohol	C_15_H_26_O	Stem and leaves (methanol extracts)	GC;GCMS;NMR	[24–27, 29–31]
*α*-, *β*-, *γ*-and *δ*-Patchoulene	C_15_H_24_	Stem and leaves (methanol extracts)	GC;GCMS;NMR	[26, 30, 31]
Pogostol	C_15_H_26_O	Leaves(steam distillation extracts)	GC;GCMS;NMR	[24, 25, 30]
Pogostone	C_12_H_16_O_4_	Stem and leaves (methanol extracts)	NMR;IR;MS;UV	[26, 30, 31]
Seychellene	C_15_H_24_	Stem and leaves (methanol extracts)	GC;GCMS;NMR	[24, 26, 27, 31, 32]
The nonvolatile constituents of *P. cablin*.				
Flavonoids				
Apigenin	C_15_H_10_O_5_	Air dried root (Ethanol extract)	HPLC	[33]
Diosmetin-7-O-*β*-D-gluco- pyranoside	C_22_H_22_O_11_	Stem (Ethanol extract)	IR;NMR	[34]
4ʹ,5-Dihydroxy-3ʹ,7- dimethoxyflavanone	C_17_H_16_O_6_	Whole plant (Ethanol & hexane extracts)	HSCCC;HPLC;NMR	[35]
5,4′-Dihydroxy-7,3′-dimethoxyflavanone	C_17_H_16_O_6_	Aerial parts (ethanol extracts)	IR;NMR;HPLC;	[36]
5,4ʹ-Dihydroxy-3,3ʹ,7- trimethoxyflavanone	C_18_H_18_O_7_	Whole plant/Root (Ethanol & hexane extracts)	HSCCC;HPLC; NMR;	[35, 37]
3,3′,4′,7-Tetramethoxy-5-hydroxyflavone	C_19_H_18_O_7_	Aerial parts (ethanol extracts)	IR;NMR;HPLC;	[36]
3,5-Dihydroxy-7,4ʹ- dimethoxyflavanone	C_29_H_36_O_15_	Whole plant/Root (Ethanol & hexane extracts)	HSCCC;HPLC; NMR;	[37]
5-Hydroxy-3,3ʹ,4ʹ,7- tetramethoxyflavone	C_19_H_18_O_7_	Stem (Ethanol extract)	NMR	[37]
5-Hydroxy-3,7,3ʹ,4ʹ- tetrmethoxyflavanone	C_19_H_ 18_O_6_	Whole plant/Root (Ethanol & hexane extracts)	HSCCC;HPLC; NMR;	[35, 37]
5-Hydroxy-7,4′-dimethoxyflavanone	C_17_H_16_O_5_	Aerial parts (ethanol extracts)	IR;NMR;HPLC;	[36]
3,5,3′,4′-Tetrahydroxy-7-methoxyflavone	C_16_H_12_O_7_	Aerial parts (ethanol extracts)	IR;NMR;HPLC;	[36]
5-Hydroxy-3,7,4′-trimethoxyflavone	C_18_H_16_O_6_	Aerial parts (ethanol extracts)	IR;NMR;HPLC;	[36]
5-Hydroxy-7,3′,4′-trimethoxyflavanone	C_18_H_18_O_6_	Aerial parts (ethanol extracts)	IR;NMR;HPLC;	[36]
Phytosterols				
Daucosterol	C_35_H_60_O_6_	Leaf/Stem (Ethanol extract)	HPLC;NMR; IR; MS;	[33]
*β*-Sitosterol	C_29_H_50_O	Dried leaves/Aerial parts (ethanol extracts)	HPLC;NMR; IR; MS;	[33, 36, 38, 39]
Stigmasterol	C_29_H_48_O	Dried leaves (Hexane extract)	GCMS	[36, 39]
Glycosides				
Agastachoside	C_24_H_24_O_11_	Stem (Ethanol extract)	IR; NMR	[34]
Apigenin-7-O- (3″, 6″-di- (E)-p- coumaroyl)-*β*-D-galacto- pyranoside	C_30_H_26_O_12_	Leaf/Stem (Ethanol extract)	IR; HPLC; NMR	[33, 34]
3*α*-Hydroxypatchoulol 3-O-*β*-D- glucopyranoside	C_21_H_36_O_7_	Air dried whole plant (Ethanol extract)	TLC	[40]
15-Hydroxypatchoulol 15-O-*β*-D-glucopyranoside	C_21_H_36_O_7_	Air dried whole plant (Ethanol extract)	TLC	[40]
3″-O-Methylcrenatoside	C_29_H_36_O_15_	Leaf/Stem (Ethanol extract)	HPLC	[33]
Soya-cerebroside I and II	C_40_H_75_NO_9_	Stem (Ethanol extract)	IR;NMR	[34]
Sesquiterpenes				
8*α*, 9*α*-Dihydroxypatchoulol	C_15_H_26_O_3_	Aerial parts (MeOH extract)	IR;NMR	[41]
3a, 8a-Dihydroxypatchoulol	C_15_H_26_O_3_	Aerial parts (MeOH extract)	IR;NMR	[41]
10*α*-Hydroperoxyguaia-1,11- diene	C_15_H_25_O_2_	Dried whole herb (Acetone extract)	IR	[42]
1*α*-Hydroperoxyguaia-10(15),11- diene	C_15_H_ 25_O_2_	Dried whole herb (Acetone extract)	IR	[42]
15*α*-Hydroperoxyguaia-1(10),11- diene	C_15_H_24_O_2_	Dried whole herb (Acetone extract)	IR	[42]
2-Keto-4*β*-hydroxyguai-1,11- diene	C_15_H_22_O_2_	Air dried stem (Ethanol extract)	UV; IR; NMR; MS	[42]
8-Keto-9(10)-*α* - patchoulene-4*α* - ol	C_15_H_22_O_2_	Air dried stem (Ethanol extract)	UV; IR; NMR; MS	[42]
6-Hydroxypatchoulol	C_15_H_26_O_2_	Aerial parts (Methanol extract)	IR;NMR	[41]
Organic Acids				
Dibutyl phthalate	C_16_H_22_O_4_	Aerial parts (Ethanol extract)	HPLC	[38]

**Table 2:** Existing research on *P. cablin*'s role in treating Intestinal mucosal inflammation.

Compound name	Animal and pathological models	Dose of intervention	Methods of administration	In vivo or in vitro	Detection indicator	Conclusion	Reference
Patchouli alcohol	Model of TNBS-induced ulcerative colitis in SD rats	0.5,15, and 30 mg/kg	Gastric lavage (medicine)	In vivo	TNF-*α*, IFN-*γ*, IL-1 *β*, IL-6, IL-17, protein of phospho-p65 and phospho-I *κ* B *α*	PA treatment reduces inflammatory responses by inhibiting the NF-*κ*B signaling pathway; PA has anti-colitis effects.	[84]
Patchouli alcohol	DSS-induced experimental colitis in C57BL/6 mice	40 mg/kg	Gastric lavage (medicine)	In vivo	IL-1*β*, IL-6, TNF-*α*, COX-2, I*κ*B*α*	PA activates PXR signaling in vitro; PA inhibits the expression of pro-inflammatory factors in THP-1 cells by inhibiting NF-*κ*B; PA ameliorates DSS-induced colitis by inhibiting inflammation in mice through mPXR/NF-*κ*B signaling	[85]
Patchouli alcohol	5-FU-induced intestinal mucositis in SD rats	PA-10, 10 mg/kg; PA-20, 20 mg/kg; PA-40, 40 mg/kg	Oral (administered by mouth)	In vivo	TNF-*α*, IL-1*β*, IL-6, MPO, IL10, TLR2,MyD88, I*κ*B*α*	PA inhibited rat intestinal mucositis, which was largely dependent on the inhibition of the TLR2/MyD88/NF-*κ*B pathway.	[86]
Patchouli alcohol	Acute liver injury model in Wistar rats	10, 20, 40 mg/kg	Gastric lavage (medicine)	In vivo	TNF-*α*, IL-1*β*, IL6, MPO, TLR4, MyD88, p-p65, p-I*κ*B*α*	PA alleviates inflammation induced by intestinal LPS leakage regulated by MyD88/TLR4/NF-*κ*B signaling pathway	[87]
Patchouli alcohol	RAW264.7 and HT-29 cells	0.1% (*v*/*v*)	—	In vitro	iNOS, IL-6, I*κ*B*α*	PA reduces inflammatory activation by inhibiting ERK-mediated NF-*κ*B activation and subsequently downregulating iNOS and IL-6 expression in mouse macrophages and human colorectal cancer cells.	[81]
Patchouli oil	5-FU-induced intestinal mucositis in SD rats	25, 50, 100 mg/kg	Oral (administered by mouth)	In vivo	MyD88, IRAK-4, FAD, TAK-1, p-p65, p65, p-p38, p38, VIP, cAMP, PKA	P.oil downregulated the expression of cytokines (TNF-*α*, IFN-*γ*, and IL-13) and inhibited the activation of NF-*κ*B and MAPK signaling.	[88]
*β*-PAE	DSS-induced experimental colitis in BALB/c mice	5, 10, 20 mg/kg	Oral (administered by mouth)	In vivo	MPO, TNF-ICAM-1, Pselectin, TLR4, MyD88	*β*-PAE significantly downregulates the TLR4/MyD88/NF-*κ*B pathway to alleviate colonic inflammation and may have TLR4 antibody-like effects.	[89]
PCW	TNBS-induced IBD in rat models	10, 50 mg/kg/d	Oral (administered by mouth)	in vivo	IL-8, MCP-1 IL-6, NF-*κ*B	PCW suppresses colonic inflammation by inhibiting the expression of NF-jB-dependent pro-inflammatory cytokines.	[90]

## Data Availability

This study is a literature review. All data cited in this article are publicly available and referenced in the manuscript.
